# Does de-implementation of low-value care impact the patient-clinician relationship? A mixed methods study

**DOI:** 10.1186/s12913-021-07345-9

**Published:** 2022-01-06

**Authors:** Michelle S. Rockwell, Kenan C. Michaels, John W. Epling

**Affiliations:** 1 Department of Family and Community Medicine, Virginia Tech Carilion School of Medicine, 1 Riverside Circle, Suite 102, Roanoke, VA 24016 USA; 2 Virginia Tech Carilion School of Medicine, 2 Riverside Circle, VA 24016 Roanoke, USA

## Abstract

**Background:**

The importance of reducing low-value care (LVC) is increasingly recognized, but the impact of de-implementation on the patient-clinician relationship is not well understood. This mixed-methods study explored the impact of LVC de-implementation on the patient-clinician relationship.

**Methods:**

Adult primary care patients from a large Virginia health system volunteered to participate in a survey (*n* = 232) or interview (*n* = 24). Participants completed the Patient-Doctor Relationship Questionnaire (PDRQ-9) after reading a vignette about a clinician declining to provide a low-value service: antibiotics for acute sinusitis (LVC-antibiotics); screening EKG (LVC-EKG); screening vitamin D test (LVC-vitamin D); or an alternate vignette about a high-value service, and imagining that their own primary care clinician had acted in the same manner. A different sample of participants was asked to imagine that their own primary care clinician did not order LVC-antibiotics or LVC-EKG and then respond to semi-structured interview questions. Outcomes data included participant demographics, PDRQ-9 scores (higher score = greater relationship integrity), and content analysis of transcribed interviews. Differences in PDRQ-9 scores were analyzed using one-way ANOVA. Data were integrated for analysis and interpretation.

**Results:**

Although participants generally agreed with the vignette narrative (not providing LVC), many demonstrated difficulty comprehending the broad concept of LVC and potential harms. The topic triggered memories of negative experiences with healthcare (typically poor-quality care, not necessarily LVC). The most common recommendation for reducing LVC was for patients to take greater responsibility for their own health. Most participants believed that their relationship with their clinician would not be negatively impacted by denial of LVC because they trusted their clinician’s guidance. Participants emphasized that trusted clinicians are those who listen to them, spend time with them, and offer understandable advice. Some felt that not providing LVC would actually increase their trust in their clinician. Similar PDRQ-9 scores were observed for LVC-antibiotics (38.9), LVC-EKG (37.5), and the alternate vignette (36.4), but LVC-vitamin D was associated with a significantly lower score (31.2) (*p* < 0.05).

**Conclusions:**

In this vignette-based study, we observed minimal impact of LVC de-implementation on the patient-clinician relationship, although service-specific differences surfaced. Further situation-based research is needed to confirm study findings.

**Supplementary Information:**

The online version contains supplementary material available at 10.1186/s12913-021-07345-9.

## Background

Low-value care (LVC) is defined as a service that offers no net benefit in specific clinical scenarios [1]. In the U.S., provision of LVC is prevalent (up to 20% of total health services), costly (approximately $30 to 100 billion annually), and has been associated with harmful outcomes for patients [2–4]. The de-implementation of LVC is increasingly prioritized in efforts to improve the quality, cost, and equity of healthcare delivery [5–7].

The American Board of Internal Medicine’s Choosing Wisely campaign and multiple other initiatives have identified hundreds of low-value services [8]. For example, annual electrocardiogram (EKG) screening in low-risk patients is associated with an expenditure of more than $40 million per year expenditure in Virginia alone [9], and may lead to costly care cascades [10, 11]. Population-based screening for vitamin D deficiency is a USPSTF D recommendation (not recommended), yet patterns of overuse are prevalent [12–14]. Low-value prescriptions for antibiotics in uncomplicated upper respiratory infection increased from 2014 to 2018 in Medicare fee-for-service patients [15] in spite of rising incidence of antibiotic-resistant infections, related mortality, and over $4 billion in annual costs to treat these infections [16, 17].

Although there is evidence of some progress in de-implementation of LVC [18], sustainable interventions to effectively reverse the trends and culture associated with LVC remain elusive [1, 15, 19]. Although many factors contribute to the provision of LVC, one commonly cited barrier to de-implementation is clinicians’ concern about negatively impacting patients’ healthcare experience [20–27]. Clinicians’ prioritization of maintaining good relationships with patients is a specific driver of LVC [20, 21, 28]. However, investigations to explore the impact of LVC de-implementation on the patient-clinician relationship are limited.

Considering the importance of the patient-clinician relationship to positive health outcomes, treatment adherence, and patient satisfaction [29–31], and the sense of urgency around LVC de-implementation, we designed a study aimed at improving understanding of patients’ reaction to LVC de-implementation, particularly as it affects the patient-clinician relationship.

## Methods

This mixed methods study aimed to 1) explore how patients respond to LVC de-implementation and 2) describe the potential impact of LVC de-implementation on the patient-clinician relationship. The study was comprised of patient interviews (qualitative) for in-depth exploration of both aims and a patient survey (quantitative) to enable more targeted focus on the patient-clinician relationship aim (Table 1). Using a convergent study design [32], the survey and interviews were conducted and analyzed in early 2021. Integration of qualitative and quantitative data was achieved at the methods and interpretation/reporting levels [33].

**Table 1:** Mixed methods study design

	**Demographics**	**Vignette^**	**Domains**
***Qualitative*** **INTERVIEW** (*n* = 24)	**Age** **Gender** **Race** **Ethnicity** **Insurer** **Education Level** **Annual Income** **Region** **PCP License**^‡^	Researchers read one vignette (randomly assigned) to each interview participant. **LVC-antibiotics** *(denied)* **LVC-EKG** *(denied)*	1. understanding of the concept of LVC 2. recognition of reasons for not providing LVC 3. the impact to the patient of not providing LVC 4. the impact to the patient-clinician relationship of not providing LVC 5. patients’ recommendations for the reduction or de-implementation of LVC
	**Demographics**	**Vignette^**	**PDRQ-9**^**37**^
***Quantitative*** **SURVEY** (n = 232)	**Age** **Gender** **Race** **Ethnicity** **Insurer** **Education Level** **Annual Income** **Region**^†^ **PCP License**^‡^	Survey respondents read one vignette (randomly assigned) at the start of the survey. **LVC-antibiotics** *(denied)* **LVC-vitamin D** ***(****denied)* **LVC-EKG** *(denied)* **HVC- statin for high CVD risk** (alternate) *(prescribed)*	You will read nine statements that a person can make about his/her primary care provider (PCP). Please choose the appropriateness of each statement for your PCP by marking one number per statement. *1 = not at all appropriate* *2 = somewhat appropriate* *3 = appropriate* *4 = mostly appropriate* *5 = totally appropriate* 1. My PCP helps me. 2. My PCP has enough time for me. 3. I trust my PCP. 4. My PCP understands me. 5. My PCP is dedicated to helping me. 6. My PCP and I agree on the nature of my medical symptoms. 7. I can talk to my PCP. 8. I feel content with my PCP’s care. 9. I find my PCP easily accessible.
***Interview and survey data were integrated for analysis and interpretation.***			

LVC-antibiotics = low-value antibiotics for antibiotics; LVC-EKG = low-value screening EKG; LVC-vitamin D = low-value screening test for vitamin D deficiency

^Vignettes are included in Supplemental Table A

^‡^PCP License = Primary Care Provider License (MD, DO, NP, PA)

The study took place within primary care of a large health system in southwest Virginia (approximately 300 clinicians, 1 million patients, and 43 practices within a 17,000 mile^2^ radius). Patients were eligible for recruitment as participants for either study if they were: 1) 18 years or older; 2) had been seen by a primary care provider in the previous 24 months; 3) were able to understand written or spoken English; and 4) had email address registered with the health system. All study procedures took place via internet, email, or telephone due to COVID-19 precautions. This research was considered exempt from human subjects review by the Institutional Review Board of Carilion Clinic. All methods were carried out in accordance with the Declaration of Helsinki.

### Interview administration

A sample of 500 eligible patients was randomly selected from the eligible population by a research analyst who was not part of the study team using a computer-generated randomization tool and stratifying only on geographic region in effort to recruit a sample representative of our health system. The patient sample received a REDCap email invitation to participate in a telephone interview about patients’ experiences with service delivery in primary care. Patients who expressed interest in participating were contacted via email to schedule a 30-min telephone interview session with a member of the research team (KM).

Interview sessions began with the interviewer reading a vignette about a patient who did not receive a particular health service - low-value antibiotics for sinusitis (LVC-antibiotics) -because the primary care clinician described such service as unnecessary and potentially harmful (Supplemental Table A). After reading the vignette to participants, the interviewer introduced semi-structured prompts encompassing the following domains:1) understanding of the concept of LVC; 2) recognition of reasons for not providing or for de-implementing LVC; 3) the impact of not providing or de-implementing LVC on the patient; 4) the impact of not providing or de-implementing LVC on the patient-clinician relationship; and 5) patients’ recommendations for the reduction of LVC.

After 12 interviews, the research team performed a preliminary review of responses to evaluate if data saturation had been reached. It was determined that saturation was achieved for all five domains, so subsequent interviews were conducted using a vignette about low-value EKG screening (LVC-EKG). Data saturation was achieved for the second vignette after 12 participants. Participants were enrolled in the study on a rolling basis (based on timing of response to the invitation to participate). Interview sessions were audio recorded, manually transcribed, and imported into Dedoose v. 8.3.4 (California, USA) qualitative analysis software. The interviewer’s written notes and reactions were also imported into Dedoose.

### Survey administration

A separate sample of 2400 patients was randomly selected from the eligible population (using methods described above) to receive a REDCap email invitation to participate in a survey about how a specific scenario may impact a patient’s relationship with their primary care provider. This randomization was stratified by geographic region of the clinic sites in attempt to acquire a sample that reflected the breadth of the health system’s primary care patient population.

Those who volunteered to participate were asked to read an informed consent statement and continue to the survey if they consented to participation. The survey first presented respondents with a single vignette (chosen at random from the set of four vignettes, Supplemental Table A) to read at their own pace. Three of the four vignettes described a clinical scenario in which a patient requested a particular low-value health service from their primary care provider but was denied that service. These services, all Choosing Wisely [8] recommendations, included: LVC-antibiotics, LVC-EKG, or a screening test for vitamin D deficiency (LVC-vitamin D). In each case, the clinician described the requested service as unnecessary based on the patient’s characteristics and health status, providing an explanation of harms that could result from receiving such service when not indicated. The fourth vignette described a similar scenario involving high-value care (HVC) in which a patient was appropriately prescribed a statin based on their clinical characteristics and cardiovascular disease risk (HVC-statin).

Following their reading of the vignette, respondents were asked to imagine that their own primary care provider had taken the action of the clinician in the vignette scenario, a procedure commonly used in the literature [34–36]. They then completed the Patient-Doctor Relationship Questionnaire (PDRQ-9) about their own provider and responded to general demographic questions. The PDRQ-9 is a brief survey instrument used to assess the quality of the patient-clinician relationship from the perspective of the patient [37]. Based on the Helping Alliance Questionnaire of Luborsky, a scale that measures therapeutic alliance in psychotherapy, the PDRQ-9 has shown high levels of reliability and validity in primary care [41]. The total PDRQ-9 score ranges from 9 to 45, with higher scores reflecting greater relationship integrity.

The survey was pilot-tested with a small group (*n* = 12) of patient respondents prior to administration. Minor modifications were made to the survey instructions and vignettes based on feedback provided. The survey required approximately 15 min to complete. A priori power analysis determined that 216 participants were needed to achieve 80% power to detect an effect size of 0.5 among the four independent vignette variables with alpha set to 0.05. Thus, we targeted 240 survey responses to account for up to 10% survey non-response. Once 240 responses were received (11 days), the survey invitation was closed to responses.

### Data analysis

#### Interview

A conventional content analysis approach was used to analyze data in Dedoose [37]. Research team members (MR, KM, and JE) independently completed an initial and thorough read of each transcript, documenting memos related to first impressions, reactions, thoughts, and questions. The research team met in person to discuss, compare, and contrast memos, and to establish an initial coding scheme. Each team member then coded transcript excerpts using the initial coding scheme, establishing additional themes and codes throughout the process. Another in-person meeting followed to merge and finalize new themes and to discuss any variance in coding and excerpts.

In addition to the content analysis, transcripts were categorized by two members of the research team (MR and KM) based on the interview participants’ responses the following questions:

-Did the interview participant comprehend the concept of LVC as related to this service? (yes, no, or maybe/unclear)
-Did the interview participant agree with the clinician’s decision to not provide or de-implement the service being discussed? (yes, no, or maybe/unclear)
-Did the interview participant indicate that not providing or de-implementing the service would impact their relationship with their primary care provider? (yes, no, or maybe/unclear)

Disagreements in categorization were discussed among the research team until a consensus was reached collaboratively. Interview data were not posted publicly to protect the privacy of participants.

#### Survey

Incomplete surveys were omitted from analyses. Descriptive characteristics (frequencies, means ± standard deviations) were calculated for demographics data and PDRQ-9 scores. One-way analysis of variance (ANOVA) with Tukey’s post-hoc test was used to evaluate differences in total PDRQ-9 scores between the four vignettes. The relationship between variables and total PDRQ-9 score was assessed via Spearman’s correlation analysis. All data were analyzed using IBM SPSS 26.0 (Illinois, USA). Significance was set at the .05 level. The full survey dataset has been posted publicly at https://osf.io/avzr5/files/.

#### Data integration

Interview and survey results were integrated and synthesized during multiple analysis sessions. The research team identified interview themes and survey results that addressed each of the two study aims (explore how patients experience LVC de-implementation and describe the impact of LVC de-implementation on the patient-clinician relationship), discussed congruence of these results, and organized on a topic-by-topic basis, using a narrative weaving approach to develop an outline for the presentation of overall findings [33].

## Results

Of the 36 patients who expressed interest in the interview, 24 were eligible, scheduled, and completed the interview. The survey was completed in full by 232 respondents (of the 240 who consented to survey participation). Demographics of the interview participants and survey respondents matched those of the overall primary care population of our health system (Table 2). There were no statistically significant differences in demographics between the four survey vignette groups (Supplemental Table B).

**Table 2:** Interview participant and survey respondent demographics

	n	Age (years)	Gender n(%)	Race n(%)	Ethnicity n(%)	Insurer n(%)	Education n(%)	Annual Income n(%)	PCP License
**Interview**	24	54.8 ± 12.2	13(54) **female**	20(83) **white**	23(96) **non-Hispanic**	14(58) **Commercial** 8(33) **Medicare** 2(9) **Medicaid** 0(0) **Other**	8(33) **< High school** 9(38) **College** 7(29) **Graduate** 0(0) **Other** 00) **Prefer to not say**	1(5) **<$25,000** 6(25) **$25 to 75,000** 13(55) **$75 to 125,000** 1(5) **$125 to 175,000** 1(5) **>$175** 1(5) **Unsure/Prefer to not say**	19(79) **MD/DO** 1(5) **NP** 4(17) **PA**
**Survey**	232 (*n* = 65) **LVC-antibiotics** (*n* = 54) **LVC-** **vitamin D** (*n* = 59) **LVC-EKG** (n = 54) **HVC-statin**	53.4 ± 15.9	151(65) **female**	216 (93) **white**	223(96) **non-Hispanic**	135(58) **Commercial** 70(30) **Medicare** 14(6) **Medicaid** 14(6) **Other**	49(21) **< High school** 104(45) **College** 72(31) **Graduate** 5(2) **Other** 5(2) **Prefer to not say**	23(10) **<$25,000** 84(36) **$25 to 75,000** 60(26) **$75 to 125,000** 21(9) **$125 to 175,000** 14(6) **>$175** 32(14) **Unsure/Prefer to not say**	167(72) **MD/DO** 49(21) **NP** 16(7) **PA**

Integrated interview and survey findings are presented below within the framework of the two study aims and four interview themes.

### Aim 1: how do patients respond to the de-implementation of low-value care?

#### Comprehension of low-value care

Of the 12 participants interviewed following LVC-antibiotics, all (12/12) expressed understanding of why the patient in the story was not prescribed an antibiotic for sinusitis that was likely caused by a viral infection. All (12/12) agreed with the clinician’s decision and agreed that they would not want to take a medication that they did not need. Most interview participants brought up the issue of widespread antibiotic resistance and their familiarity with goals to reduce overuse of these medications. However, three interview participants described feeling that their intuition is often a better indicator of when an antibiotic is needed than their primary care provider’s. Thus, these participants have “*asked until he caves*” (PT13) or gone to an urgent care provider or emergency department where they were ultimately prescribed antibiotics.

All participants (12/12) interviewed following LVC-EKG expressed at least some level of understanding about why the clinician in the story did not order a screening EKG. Some interview participants (5/12) did not agree with the clinician’s decision, stating that ordering an EKG “just in case” seemed like a reasonable or preferable option, especially if insurance covered the service or if it was important to the patient. In general, interview participants were unable to imagine potential harm that may arise from LVC-EKG, other than unexpected costs.

*“I just don’t see the harm in it. If a person has insurance and insurance is going to cover it and I can make my copay, what’s the harm in it?”* (PT18)

*“I think it’s more based on patient preference, like if the patient is pressing it…that’s when I would go ahead and get one done…just as a precaution.”* (PT21)

**Fig. 1:**
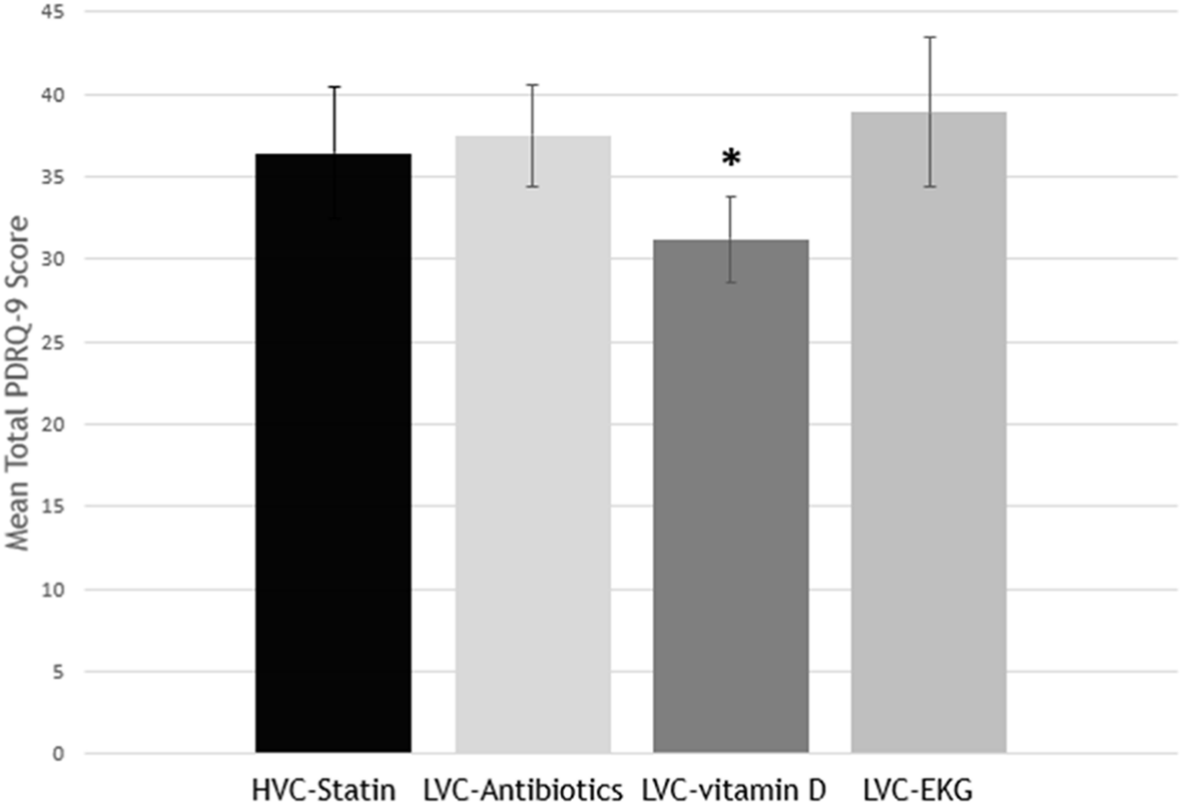
Patient-Doctor Relationship Questionnaire (PDRQ-9) Results by Vignette. Possible PDRQ-9 total score range = 9 to 45, with higher score reflecting greater relationship integrity. *p < 0.05

Some evidence of service-specific differences in patients’ experience with LVC (as related to the patient-clinician relationship) was also observed in the survey findings. The mean total PDRQ-9 score for LVC-vitamin D was significantly lower than the other vignettes (Fig. 1). Lower individual responses to PDRQ-9 questions #3-I trust my PCP; #4-My PCP understands me; and #6-My PCP and I agree on the nature of my medical symptoms in LVC-vitamin D drove the difference in total score compared with the other vignettes. There was no difference in mean total PDRQ-9 scores between LVC-antibiotics, LVC-EKG, and HVC-statin.

Although interview participants recognized the two vignette scenarios as LVC, there was little evidence that LVC was viewed as a broad problem. A few cited overall healthcare costs related to LVC as a concern and three acknowledged risk of false positive test results and some demonstrated understanding of downstream consequences and cascades that may result from provision of LVC.

*“…they try every test under the sun and at the end, they almost always turn out the same way every time…I guess that’s what they are programmed to do. It’s an example of overkill…and in one case, he got an incorrect diagnosis and the trail of new problems and test started from there…but none that actually helped.”* (PT14)

*“A lot of times with elderly people…they would send for an ambulance and take her to the emergency room and then this whole cascade of tests started, you know…but at some point…there is the law of diminishing returns on bringing out the full battalion or whatever.”* (PT17)

For the most part, interview participants perceived underuse of health services as a bigger issue than overuse. Several interview participants rationalized the need for more screenings and tests based on consumers “deserving” or clinicians “needing” data to make accurate diagnoses and treatment plans.

#### Thoughts, feelings, and memories triggered by the topic of low-value care

The vignettes and open-ended interview questions about LVC seemed to trigger thoughts, feelings, and memories related to 1) participants’ previous negative experiences with healthcare and 2) participants’ trust in their primary care clinician.

Some of the negative experiences shared by interview participants illustrated LVC, particularly related to concern about opioid over-prescribing. For example, one participant described being prescribed “*a couple months’ worth of painkillers*” (PT04) after surgeries for which he felt he only needed aspirin to control his pain. Most negative experiences shared, however, were not examples of LVC. Rather, they describe care perceived as poor-quality by the participant. Participants shared multiple examples of care they felt was ineffective, too slow, or inequitable. Others shared examples of errors, such as missed diagnoses or improperly dosed medications.

Aspects of the delivery of care were also described negatively. Some interview participants described feeling rushed, unheard, minimized, not taken seriously, or not receiving the style of treatment they expected from clinicians. Many participants expressed frustration about clinicians not explaining things in a way that they could understand or failing to take time to clearly explain a diagnosis or treatment.

*“There’s a certain amount of time that physicians are kind of supposed to spend with their patients. It’s like there’s a countdown when you enter the room, I wish they would stop that…it makes people just feel like a number…herded in and herded out.”* (PT05)

In contrast, most interview participants, including the majority who described negative experiences, volunteered information about the high trust they place in their primary care clinician.

*“…my physician is a very thoughtful and brilliant doctor, and I think she’s trying to do a good job for me. That’s her whole mission in life, I can just tell…I trust her entirely and she takes care of me.”* (PT14)

*“He listens and to tell you the truth, he knows more about me, more personal things, than literally anyone else in my life. I trust him is why…I trust him more than most anyone I know.”* (PT19)

*I think that trust and confidence that your PCP really listens and knows you matters. It’s a rather intimate process if it’s done right.”* (PT15)

*“[My doctors] are people I can trust and I do trust. That is good fortune to have in your life.”* (PT16)

*“It is extremely reassuring to have the trust in your provider that I have in my doctor.”* (PT11)

Clinician characteristics associated with high trust included good listening skills, a kind and non-rushed demeanor, treating patients in a non-condescending manner, being similar to patients (culturally or in age), using language that patients understand, and being willing to spend time with patients. Longevity of care was also important to interview participants; several described their trust being rooted in a more than 30-year relationship with their primary care clinician and others reported distress when former long-term providers had retired.

#### Recommendations for reducing the utilization of low-value care

Many interview participants said they did not have any recommendations for steps that clinicians or patients could take to reduce LVC. For some this seemed to be because the concept of LVC was not well-understood. Others stated that financial incentives inherent in fee-for-service healthcare and “*the litigious society in which we live*” (PT06) as significant obstacles.

The most common recommendation was for patients to take a more active role in their health and healthcare. Several participants suggested that patients speak up more candidly to their clinicians, be honest, ask questions, attend appointments regularly, and make lifestyle changes (especially diet and exercise) to improve health and “*prevent the need for tests and services in the first place*” (PT13). Other recommendations included educating patients, providing reputable resources to patients, and fostering a strong patient-clinician relationship.

### Aim 2: does low-value care de-implementation impact the patient-clinician relationship?

Total score on the PDRQ-9 survey for the three LVC vignettes was 35.9 ± 4.9 out of 45 possible points. This total score equates to a mean of 3.99 for individual responses (a response of 4 = “mostly appropriate” for the PDRQ-9 statements shown in Table 1). The complete survey dataset has been posted at https://osf.io/avzr5/files/.

Of the interview participants, 17/24 believed that their relationship with their primary care clinician would not be impacted negatively by not receiving the service described in the vignette, while 4/24 believed their relationship would be impacted negatively, and 3/24 were unsure. A strong relationship with their clinician, trust in their expertise and character, and faith in their guidance were cited as justification by interview participants who did not believe their relationship would be negatively impacted.

*“I’ve had that issue with a medication before, when the doctor and me did not agree on which medication to go on. We actually went with the one they recommended because I figure I have been with him awhile and trust him so kindof sensed that their judgement should be over mine.”* (PT21)

*“My doctor would never do that but other doctors don’t think about how the outcome will be for their patients.”* (PT19)

Some (*n* = 8) even felt that not providing the service would increase their satisfaction and trust in their clinician.

*“Actually, if my provider lets me know that a service isn’t really necessary for me, it would enhance my trust in her.”* (PT22)

*“There's rules that the doctors have to go by as well, as far as giving good care. This includes rules for not overprescribing and doing too much…I wouldn't be happy with that doctor. I want a doctor who follows the rules.”* (PT03)

Interview participants who believed their relationship may be negatively impacted seemed to recognize the complexity of the scenario, discussing complex patients, medical necessity, potential harm, insurance, and the option to go elsewhere (ex: urgent care or a new doctor). Four participants volunteered their experiences with not receiving prostate-specific antigen (PSA) testing as part of their annual physical examinations as they had in previous years. Each described the difficulty they had experienced with accepting this change, which they noted could be challenging for clinicians to navigate. Some participants stated that, in some cases, no amount of health services would feel like too much, particularly for “*serious things like cancer or your heart*” (PT11). Most participants emphasized that negative impacts could be mitigated if the clinician listened to them, spent time with them, and offered understandable advice.

*“The patient said she would be lying if she said it didn’t [impact trust]. She had a recent issue related to this and now realizes her physician was making the better recommendation. She has used it as a learning experience.”* (KM, interview notes)

*“I wanted a particular procedure. And the doctor said - No, this one's better for you and here's why…And he could explain to me why it wasn’t a good option. Even though I was irritated to begin with, when I sat back and thought about it and did a little more research, I said ‘Okay, he's right.’ I got right over myself.”* (PT13)

A number of interview participants speculated that younger patients’ relationship with their clinician may be impacted more than that of older patients since they may feel more empowered in navigating their care and because “*young folks are less cultured to keep their mouths shut and just do what the doctor says*” (PT24). However, there was no difference in mean total PDRQ-9 survey score based on age, nor gender, race, ethnicity, insurer, annual income, region, or primary care provider license. There was a statistically significant, but weak, positive correlation between education and total PDRQ-9 score (r = 0.2, *p* < 0.01). (Supplemental Table C).

## Discussion

The prevalence of LVC that currently exists in the U.S. is unsustainable. Clinicians and other stakeholders perceive negative impacts to patients as a barrier to de-implementing LVC. Some [26, 38], but not all [39], previous research has shown diminished patient satisfaction ratings when LVC was not provided. However, the impact of LVC de-implementation on the patient-clinician relationship is unknown. In this mixed methods exploration into the patient experience with LVC de-implementation, we observed minimal evidence of negative impact to the patient-clinician relationship. Total PDRQ-9 survey scores were similar to (± 10%) [40–42] or higher than [43] those reported by other investigators. Likewise, few interview participants believed that their relationship with their clinician would be negatively impacted by not receiving LVC.

The topic of trust emerged repeatedly throughout the present study. The majority of participants described high levels of trust in their primary care clinician and their reliance on this trust when they encountered health concerns or problems with navigating their healthcare. However, the PDRQ-9 question most reduced with the LVC-vitamin D vignette was #3-I trust my PCP, indicating the potential sensitivity of trust to de-implementation of some low-value services. Given the influence of trust to a strong patient-clinician relationship [30] and evidence of decreasing trust in healthcare and healthcare clinicians in the U.S. [44], further research into the impact of LVC de-implementation on multi-level patient trust is warranted. Additionally, multiple participants suggested LVC de-implementation as a potential enhancer of trust in their clinician, which conflicts with commonly reported clinician concerns [20, 24, 26]. Investigations to explore the framing of LVC de-implementation as a means of enhancing trust are needed.

A recent meta-analysis found that de-implementation interventions that engage patients in in the context of the patient-clinician interaction are effective in reducing the utilization of LVC [45]. Some participants in the present study, and those in other investigations [45, 46], reported high readiness to play a role in LVC de-implementation. However, we also observed some difficulty in understanding the concept of LVC, imagining harms that may arise from the receipt of LVC, and in perceiving LVC as a broad problem. Unfortunately, the topic of LVC elicited memories and stories about poor-quality care or bad experiences with the healthcare system, which may provide additional evidence that the LVC concept is not well-understood. Education and tailored messaging will be important components of patient-centered de-implementation interventions.

The impact of LVC de-implementation on the patient experience may be service-specific, as observed in the present study. While this finding may be associated with the popularity of vitamin D in the scientific literature and popular media [47], further research on service-level variation in patient-reported outcomes in response to de-implementation is needed.

We observed no differences in patient response based on demographic factors, except for education level. Respondents who had experienced lower levels of education reported a greater impact of not receiving a service on their relationship with their clinician. Others have reported disparities in the provision of and de-implementation of LVC based on factors such as region and patient race [4, 48].

### Limitations

There are some limitations to this study, mostly related to the use of vignettes. Vignettes are widely used in health services research, and have been shown to reliably represent patients’ clinical experiences, behaviors, and perspectives [49]. An assumption of the study design was that participants were able to imagine themselves in an exchange with their own PCP similar to the one described in the vignettes. This approach was deliberate as previous research has shown hypothetical situational vignettes as effective in the stimulation and elicitation of focused reflection and has been used throughout the health services literature. The vignettes also (intentionally) presented scenarios as patient requests for services that the clinician denied based on being categorized as LVC. Although patient request or demand for LVC is common [21, 22], there are other drivers of LVC utilization. Finally, many real-world patient-clinician interactions around LVC are brief in duration. The fact that patients had a more lengthy interview period (up to 30 min) to consider their reactions may have influenced their responses.

We only invited continuous primary care patients with an email address on file to participate in the study, which may have biased the cohort in ways that are unpredictable. However, since demographic characteristics of interview participants and survey respondents very closely matched those of the overall primary care population, we assume the cohorts were representative of the overall primary care patient population. Finally, the study took place in a single health system in one region of the U.S. Although the footprint of this health system spans more than one-quarter of the state of Virginia, representing both rural and urban regions and a diversity of patients, it is unclear if results are generalizable to other regions. There are numerous opportunities to build upon the novel findings of this study.

## Conclusions

Among primary care patients, we observed varied understanding of the concept and consequences of LVC. Although the topic stimulated discussion about trust in the patient-clinician relationship, it also triggered memories of poor-quality care and negative encounters with healthcare. In this vignette-based study, we observed minimal evidence that de-implementation of low-value antibiotic prescribing or EKG screening would negatively impact the patient-clinician relationship. In fact, some participants associated de-implementation with *increased* trust in their clinician. A clinician’s refusal to order a low-value vitamin D screening test, however, elicited a greater negative impact, suggesting that effects of de-implementation may vary by service. Clinicians commonly cite concern about patient satisfaction ratings and the integrity of the patient-clinician relationship as barriers to the de-implementation of LVC. However, our results suggest that in the presence of strong patient-clinician communication, trust, and understanding, patients stand ready to play a role in reducing LVC. Further research is needed to confirm these findings in real-world clinical scenarios.

## Supplementary Information

**Additional file 1.**

## Data Availability

The survey dataset generated during the current study are available at https://osf.io/avzr5/files/. The interview transcripts from the current study are not available to protect the privacy of participants and their providers.

## Abbreviations

USPSTF: US Preventive Services Task Force
EKG: electrocardiogram
LVC: low-value care
HVC: high-value care
LVC-antibiotics: vignette about low-value antibiotics prescribing
LVC-vitamin D: vignette about low-value vitamin D screening
LVC-EKG: vignette about low-value electrocardiogram screening
HVC-statin: vignette about high-value statin prescribing
PDRQ-9: Patient-Doctor Relationship Questionnaire-9 questions
PSA: prostate-specific antigen
