# Supplementary material for: Does de-implementation of low-value care impact the patient-clinician relationship? A mixed methods study
Source: BMC Health Serv Res. 2022 Jan 6;22:37. doi: 10.1186/s12913-021-07345-9 (PMC8733793; doi:10.1186/s12913-021-07345-9)
Supplement: Supplementary file 1 — Additional file 1. [file 12913_2021_7345_MOESM1_ESM.docx]

**Supplemental Table A. Study Vignettes**

1. **Low-value Antibiotics for Sinusitis (LVC-antibiotics)**

Taylor makes an appointment with her primary care physician, Dr. Jones, because she is not feeling well. She has a headache, pressure in her face, and a sore throat.

After talking with and examining her, Dr. Jones explains that she likely has “acute sinusitis” – also known as a sinus infection. Taylor asks for a prescription for antibiotics. Dr. Jones does not prescribe antibiotics for Taylor since he determines that Taylor’s sinus infection is caused by a virus. He explains that antibiotics are effective medications for treating infections caused by bacteria, but not for those caused by viruses.

Dr. Jones also shares that taking unnecessary antibiotics may also be harmful since they can cause side effects and lead to antimicrobial resistance in the population. Antimicrobial resistance is when bacteria stop responding to the antibiotics designed to destroy them. Dr. Jones provides some other recommendations to address Taylor’s symptoms from the sinus infection.

**Choosing Wisely Recommendation (American Academy of Family Physicians): *Don’t routinely prescribe antibiotics for acute mild-to-moderate sinusitis unless symptoms last for ten or more days, or symptoms worsen after initial clinical improvement.***

[**https://www.choosingwisely.org/clinician-lists/american-academy-family-physicians-antibiotics-for-sinusitis/**](https://www.choosingwisely.org/clinician-lists/american-academy-family-physicians-antibiotics-for-sinusitis/)

1. **Low-value Vitamin D Screening (LVC-vitamin D)**

Taylor makes an appointment with her primary care physician, Dr. Jones, for a routine checkup.

Taylor recently read an article online that said that low levels of vitamin D are associated with cancer and heart disease. She asks Dr. Jones to order a blood test to measure her vitamin D levels. After talking with and examining her, Dr. Jones does not order a blood test to measure Taylor’s vitamin D levels. He explains that vitamin D is an important nutrient for health, but that the medical guidelines do not indicate that testing vitamin D levels is appropriate.

Dr. Jones shares that performing a vitamin D blood test that is not medically needed may also be harmful to patients. For example, going to the lab to have a blood test can be inconvenient, time-consuming, and expose patients to illnesses. Additionally, Vitamin D blood tests may be expensive. Finally, there is no evidence that testing for vitamin D levels improves health. Dr. Jones recommends specific foods high in vitamin D and sensible sun exposure to ensure an adequate amount of vitamin D for Taylor’s body’s needs.

**Choosing Wisely Recommendation (American Society for Clinical Pathlology): *Don’t perform population-based screening for 25-OH-Vitamin D deficiency.***

[**https://www.choosingwisely.org/clinician-lists/american-society-clinical-pathology-population-based-screening-for-vitamin-d-deficiency/**](https://www.choosingwisely.org/clinician-lists/american-society-clinical-pathology-population-based-screening-for-vitamin-d-deficiency/)

1. **Low-value EKG Screening (LVC-EKG)**

Taylor has an appointment for an annual exam with her primary care physician, Dr. Jones. She has been feeling well and has no specific complaints.

Taylor’s friend told her that when she had her annual exam, her physician ordered an electrocardiogram (EKG) to make sure that her heart is healthy. Since she believes this test may be good for her as well, Taylor asks Dr. Jones to order an EKG. After talking with and examining her, Dr. Jones does not order an EKG for Taylor. He explains that EKGs and other cardiac screening tests are important for patients with certain physical symptoms or risk factors for heart disease, but that since Taylor does not have these symptoms or risk factors, the test is not needed for her.

Dr. Jones shares that performing an EKG that is not medically needed may also be harmful to patients. For example, going to the lab to have the test can be expensive, inconvenient, time-consuming, and expose patients to stress and illness. Also, test results may be unclear, which may lead to the ordering of additional tests or medications that have risks. Dr. Jones provides recommendations for Taylor to help keep her heart healthy.

**Choosing Wisely Recommendation (American Academy of Family Physicians): *Don’t order annual electrocardiograms (EKGs) or any other cardiac screening for low-risk patients without symptoms.***

[**https://www.choosingwisely.org/clinician-lists/american-academy-family-physicians-annual-electrocardiograms/**](https://www.choosingwisely.org/clinician-lists/american-academy-family-physicians-annual-electrocardiograms/)

1. **High-value Statin Use (HVC-statin) -- *alternate***

Taylor has an appointment with her primary care physician, Dr. Jones, to review some lab work results she had done at her annual check-up appointment.

Taylor communicates to Dr. Jones that she wants to do everything she can to keep her heart healthy. Dr. Jones shows Taylor a calculation based on her cholesterol results, her age, blood pressure history, and other heart disease risk factors, which reveals that she has a 15% risk of heart disease over the next 10 years. All of the major guidelines about heart disease recommend statin medications at Taylor’s risk level. Based on this, Dr. Jones recommends a statin medication to take daily to help reduce her risk.

Dr. Jones explains the major and minor side effects associated with statins.  The major side effects (muscle and kidney damage) are very rare, and the minor side effects (muscle aches only) are a little more common, but are reversible when the medication is stopped.  The medication is covered by Taylor’s insurance.

**US Preventive Services Task Force Recommendation: Adults without a history of cardiovascular disease (CVD) (ie, symptomatic coronary artery disease or ischemic stroke) use a low- to moderate-dose statin for the prevention of CVD events and mortality when all of the following criteria are met: 1) they are aged 40 to 75 years; 2) they have 1 or more CVD risk factors (ie, dyslipidemia, diabetes, hypertension, or smoking); and 3) they have a calculated 10-year risk of a cardiovascular event of 10% or greater.**

[**https://www.uspreventiveservicestaskforce.org/uspstf/recommendation/statin-use-in-adults-preventive-medication**](https://www.uspreventiveservicestaskforce.org/uspstf/recommendation/statin-use-in-adults-preventive-medication)

**Supplemental Table B. Demographic Characteristics of Survey Respondents by Group (Vignette**

| **Vignette** | **n** | **Gender**  **n (%)** | **Age**  **(years + SD)** | **Race**  **n (%)** | **Ethnicity**  **n (%)** | **Insurer**  n (%) | **Education**  n (%) | **Annual Income**  n (%) |
| --- | --- | --- | --- | --- | --- | --- | --- | --- |
| LVC-antibiotics | 65 | 43(66) female | 52.9 + 10.1 | 2(3) Black  62(95) White  1(2) other | 2(3) Hispanic  63(97) Non-Hispanic | 34(52) Commercial  3(5) Medicaid  22(34) Medicare  6(9) other | 15(23) High School  27(42) College  17(26) Graduate/Prof.  3(5) other  3(5) prefer not to say | 6(9)<25K  23(35) 25 to 75K  17(26) 75 to 125K  5(8) 125 to 175K  6(9)>175K  8(12) prefer not to say |
| LVC-vitamin D | 54 | 38(70) female | 53.8 + 15.7 | 2(4) Black  49(90) White  3(6) other | 3(6) Hispanic  51(94) Non-Hispanic | 34(63) Commercial  4(7) Medicaid  15(28) Medicare  1(2) other | 11(20) High School  23(43) College  19(35) Graduate/Prof.  0(0) other  1(2) prefer not to say | 5(9)<25K  20(27) 25 to 75K  14(26) 75 to 125K  4(7) 125 to 175K  2(4) >175K  9(17) prefer not to say |
| LVC-EKG | 59 | 36(61) female | 54.9 + 17.2 | 1(2) Asian  2(3) Black  53(90) White  3(5) other | 3(5) Hispanic  56(95) Non-Hispanic | 31(53) Commercial  4(7) Medicaid  18(31) Medicare  6(10) other | 12(20) High School  28(47) College  17(29) Graduate/Prof.  2(3) other  0(0) prefer not to say | 7(12)<25K  23(39) 25 to 75K  16(27) 75 to 125K  6(10) 125 to 175K  0(0) >175K  7(12) prefer not to say |
| HVC-statin | 54 | 34(63) female | 52.0 + 16.9 | 2(4) Black  52(96) White | 2(4) Hispanic  52(96) Non-Hispanic | 36(67) Commercial  3(6) Medicaid  15(28) Medicare  0(0) other | 10(19) High School  25(46) College  18(33) Graduate/Prof.  0(0) other  1(2) prefer not to say | 5(9)<25K  17(31) 25 to 75K  15(28) 75 to 125K  6(11) 125 to 175K  3(6) >175K  8(13) prefer not to say |
| Overall | 232 | 151(65) female | 53.4 + 15.9 | 1(1) Asian  8(3) Black  216(93) White  7(3) Other | 10(4) Hispanic  222(96) Non-Hispanic | 135(58) Commercial  14(6) Medicaid  70(30) Medicare  13(6) other | 48(21) High School  103(45) College  71(31) Graduate/Prof.  5(2)Other  5(2) prefer not to say | 23(10) <25K  83(36) 25 to 75K  60(26) 75 to 125K  21(9) 125 to 175K  13(6) >175K  32(14)prefer not to say |

LVC-antibiotics = low-value antibiotics for antibiotics; LVC-EKG = low-value screening EKG; LVC-vitamin D = low-value screening test for vitamin D deficiency

**Supplemental Table C. Correlation Table**

|  | | | **PDRQ-9** | **age** | **gender** | **race** | **ethnicity** | **education** | **insurance** | **income** | |  |
| --- | --- | --- | --- | --- | --- | --- | --- | --- | --- | --- | --- | --- |
|  | **PDRQ-9 score** | Correlation Coefficient (r) | 1.000 | -.003 | -.043 | .014 | -.025 | .230^**^ | .075 | .086 |  |  |
|  |  | P value | . | .961 | .517 | .827 | .701 | .006 | .253 | .193 |  |  |
|  | **age** | Correlation Coefficient (r) | -.003 | 1.000 | -.222 | .030 | -.039 | -.026 | .550^**^ | .106 |  |  |
|  |  | P value | .961 | . | .101 | .650 | .557 | .694 | .000 | .108 |  |  |
|  | **gender** | Correlation Coefficient (r) | -.043 | -.222 | 1.000 | -.001 | -.023 | -.006 | -.116 | -.085 |  |  |
|  |  | P value | .517 | .101 | . | .993 | .727 | .929 | .077 | .196 |  |  |
|  | **race** | Correlation Coefficient (r) | .014 | .030 | -.001 | 1.000 | .329^**^ | .120 | .022 | -.054 |  |  |
|  |  | P value | .827 | .650 | .993 | . | .000 | .068 | .736 | .415 |  |  |
|  | **ethnicity** | Correlation Coefficient (r) | -.025 | -.039 | -.023 | .329^**^ | 1.000 | -.006 | .051 | -.040 |  |  |
|  |  | P value | .701 | .557 | .727 | .000 | . | .923 | .436 | .544 |  |  |
|  | **education** | Correlation Coefficient (r) | .230^**^ | -.026 | -.006 | .120 | -.006 | 1.000 | -.118 | .296^**^ |  |  |
|  |  | P value | .006 | .694 | .929 | .068 | .923 | . | .074 | .000 | | |
|  | **insurance** | Correlation Coefficient (r) | .075 | .550^**^ | -.116 | .022 | .051 | -.118 | 1.000 | -.099 | | |
|  |  | P value | .253 | .000 | .077 | .736 | .436 | .074 | . | .133 | | |
|  | **income** | Correlation Coefficient (r) | .086 | .106 | -.085 | -.054 | -.040 | .296^**^ | -.099 | 1.000 | | |
|  |  | P value | .193 | .108 | .196 | .415 | .544 | .000 | .133 | . | | |

Spearman’s Correlation analysis results.

**= p<0.01
